# Geriatric Chest Imaging: When and How to Image the Elderly Lung, Age-Related Changes, and Common Pathologies

**DOI:** 10.1155/2013/584793

**Published:** 2013-07-01

**Authors:** J. Gossner, R. Nau

**Affiliations:** ^1^Department of Clinical Radiology, Göttingen-Weende Hospital, An der Lutter 24, 37074 Göttingen, Germany; ^2^Department of Geriatric Medicine, Göttingen-Weende Hospital, An der Lutter 24, 37074 Göttingen, Germany

## Abstract

Even in a global perspective, societies are getting older. We think that diagnostic lung imaging of older patients requires special knowledge. Imaging strategies have to be adjusted to the needs of frail patients, for example, immobility, impossibility for long breath holds, renal insufficiency, or poor peripheral venous access. Beside conventional radiography, modern multislice computed tomography is the method of choice in lung imaging. It is especially important to separate the process of ageing from the disease itself. Pathologies with a special relevance for the elderly patient are discussed in detail: pneumonia, aspiration pneumonia, congestive heart failure, chronic obstructive pulmonary disease, the problem of overlapping heart failure and chronic obstructive pulmonary disease, pulmonary drug toxicity, incidental pulmonary embolism pulmonary nodules, and thoracic trauma.

## 1. Introduction

The population in many societies is getting older. The United Nations estimate that the number of people older than 65 years will increase from 743 million in 2009 to 2 billions in 2050. At this time, there will be more people older than 65 years than children younger than 15 years [1]. In fact, around 15% of patients treated in German hospitals are already older than 80 years [2]. With age, the frequency of multimorbidity increases. Geriatric medicine uses the term frailty to describe the process of progredient loss of mental and physical performance making the patients more vulnerable to further disease [3]. Sometimes it is difficult to separate the process of ageing from disease itself. Therefore, diagnostic imaging of older patients requires special knowledge. In this review, after a short description of imaging strategies, ethical considerations, and the normal ageing processes of the lung, distinct pathologies with a special relevance for the elderly patient are discussed. 

## 2. Imaging Strategies

In contrast to younger people, handling of elderly patients is different and usually takes more time. In most cases, elderly patients have to be transferred to the radiology department and may need supervision while waiting. Positioning requires more time, and often patients need assistance. With bedridden patients, more than one person is needed for proper positioning. This need for more time and staff has to be kept in mind but is in most cases not reimbursed [4]. The ideal imaging test for elderly patient is fast and needs few changes in positioning. 

### 2.1. Chest Radiography

The standard examination in imaging of the lung is chest radiography with a posterior-anterior and a lateral projection. Chest radiography is easy to perform, cheap, and, according to the ACR Appropriateness Criteria, in most scenarios the initial test when lung disease is suspected [5]. In frail patients, standard projections of the chest often cannot be obtained, and a chest radiograph in supine position has to be taken with well-known limitations. 

### 2.2. Computed Tomography

**Figure 1:**
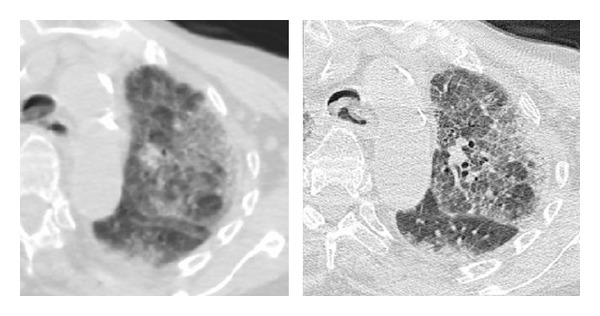
Motion artifacts due to breathing in an elderly patient impairing interpretation of the interstitial changes. An additional scan with the use of a standard high resolution technique is substantially improving diagnostic performance.

In addition to conventional X-ray, the ideal test in more complex cases is computed tomography (CT). With modern multislice CT scanners, the lung can be examined in a few seconds. But even with modern CT scanners, motion artifacts due to breathing may be a problem in the elderly. Strategies to reduce these motion artifacts include the caudal start of the scan, where motion artifacts due to breathing are pronounced, and the use of a higher pitch. If there are still marked motion artifacts causing problems with image interpretation, we are adding several axial slices in classical high resolution CT technique (Figure 1). 

Imaging of the lung parenchyma is possible without contrast media, but for imaging of lung vessels with computed tomographic pulmonary angiography (CTPA) or tumor staging, contrast media are mandatory. Elderly patients are at higher risk for contrast medium-induced nephropathy (CIN). In some cases, renal function is already impaired and there are other risk factors like diabetes, high blood pressure, heart insufficiency, hypovolemia, and atherosclerosis. Age > 75 years is also an independent risk factor for CIN [6]. It has to be considered, however, that only a very small part of patients with CIN require hemodialysis [6]. The most important prophylaxis is adequate hydration, which is especially important in elderly patients who often drink too little. The incidence of CIN is also related to the amount of used contrast media [7]. Interestingly, it has been reported that even thoracic CT scans with 15 mL iodinated contrast media showed satisfactory diagnostic quality for routine chest scans, for example, in the staging of mediastinal lymph nodes [8]. CTPA requires optimal opacification of the lung vessels, and therefore a minimum of 60 mL contrast media should be used. A high flow rate is usually recommended to obtain a good vascular enhancement. Unfortunately, poor peripheral venous access is common in the elderly and sometimes only small bore cannulas can be placed. We have shown that even with low flow rates (2.0 or 2.5 mL/s) and 60 mL of contrast media, sufficient vascular enhancement can be obtained [9]. Another strategy to minimize the dose of contrast media is the use of low kV settings [10]. 

### 2.3. Transthoracic Ultrasound

Transthoracic ultrasound may offer additional information to conventional chest radiography [11]. In the frail patient, it is easy to use bedside test. In the case of pleural effusion, it is more sensitive than radiography (especially compared to supine radiographs) and adds further information about the composition of the pleural fluid [11]. For example, it may show septations, a major cause for insufficient drainage after pleural tube placement. With transthoracic ultrasound, further information about congestive heart failure, pneumothorax, or pleura-based consolidations can be obtained [11]. 

### 2.4. Nuclear Medicine

Ventilation-perfusion scintigraphy has been replaced in the imaging of acute pulmonary embolism (PE) by CTPA in most departments. In elderly patients, prevalence of existing lung disease and therefore abnormal chest X-ray is relatively high, and therefore in this patient group, the sensitivity for scintigraphy in diagnosing PE is impaired [12]. In the elderly patient, CT scanning should be the preferred test, especially as in a substantial number of patients, diagnosis other than PE can be found (pneumonia, congestive heart failure). In patients with known allergy to iodinated contrast media, nephropathy or manifest hyperthyroidism scintigraphy is a good alternative. 

### 2.5. Magnet Resonance Imaging (MRI)

Although recent advances with lung MRI are not used in everyday imaging of the lung, an exception is the detailed imaging in Pancoast tumors. If lung imaging with MRI is performed in the elderly, a comprehensive examination to avoid excessive examination times should be used, for example, a combination of fast spin echo T2-weighted coronal images and diffusion weighted images [13]. With poor renal function, the use of contrast media is also of concern because of possible nephrogenic systemic fibrosis. A lot of older patients have contraindications for MRI like cardiac pace makers or older ferromagnetic surgical material. 

### 2.6. A Practical Approach of Lung Imaging in the Elderly

**Scheme 1:**
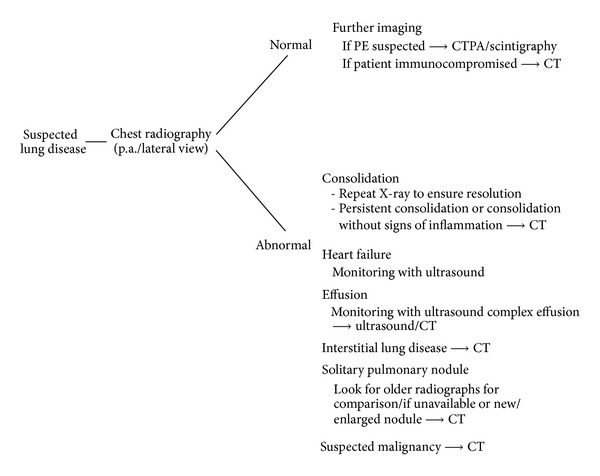


The basic examination is chest radiography. If further workup is needed (suspected pulmonary embolism, immunocompromised patient, consolidation without clinical or laboratory signs of inflammation, and mass or complex effusion), a chest CT should be performed. A thin collimated scan (1 mm slice thickness) in caudocranial direction is recommended. Contrast media should be administered only if necessary. In the followup, the modality should be chosen conidering the underlying disease. A possible imaging algorhithm is provided in Scheme 1.

## 3. Ethical Considerations

Ethical considerations are important in the care of the elderly. Treating physicians always need to consider if a potential diagnosis obtained by imaging may alter treatment. Otherwise, the test should not be done. Even in cases where an exact diagnosis, for example, staging in a malignant disease, may help to plan further optimal treatment, it has to be accepted that patients may refuse imaging and insist on palliation only towards the end of their life. In particular, elderly patients should not feel to be obliged to agree with further diagnostics [14]. 

## 4. Changes of the Lungs with Ageing

**Figure 2:**
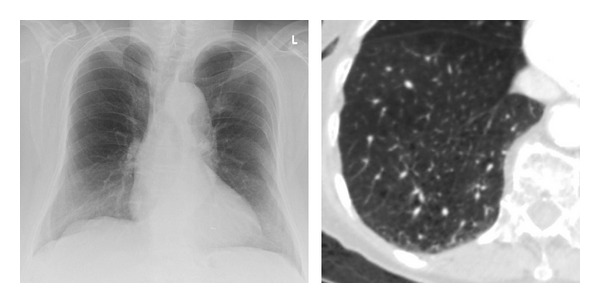
Senile emphysema in an 88-year-old patient. Chest X-ray (on the left) and centrilobular emphysematous changes on computed tomography (on the right). Imaging was ordered because of suspected mesenterial ischemia.

With ageing, an enlargement of the distal airspaces due to the loss of supporting tissue can be found, a condition for which the terms “senile lung,” “senile hyperinflation,” or “senile emphysema” have been proposed [15, 16] (Figure 2). 

**Figure 3:**
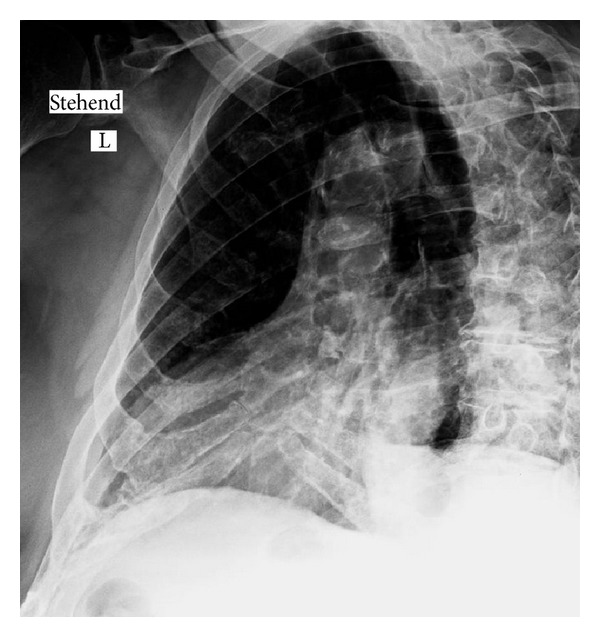
Extensive calcifications of the cartilaginous parts of the rib cage in an 85-year-old patient (suspected fracture).

Histologically, a homogeneous dilatation of the airspaces without signs of inflammation, fibrosis, or other architectural distortions can be seen [15]. As a result, signs of hyperinflation can be seen on conventional chest radiography [17]. In a recent study, there were more elderly asymptomatic adults (age > 75 years) with centrilobular emphysematous changes in CT imaging compared to a younger control group (age < 55 years) [18]. In the same study, interstitial changes of the lung with a subpleural reticular pattern could be found in 60% of the elderly patients. Bronchial wall thickening was shown in 50%. In another study, 25% of the elderly asymptomatic patients showed small cysts [17]. Lee et al. showed an increased air trapping with age [19]. The frequent finding of small basal atelectasis in asymptomatic elderly patients has been reported [20]. Further morphological changes with ageing are progressive calcifications of the airways and the rib cage [15, 16, 20] (Figure 3). 

Like other muscles, there is a loss of diaphragmatic muscle mass [16], but in an older CT study, no measurable decrease in muscle thickness of the diaphragm could be found [21]. The reduced mass of other thoracic muscles has been described but has not been studied in detail [20].

## 5. Borderlands of the Normal: Possible Problems in Differentiation between Age-Related Changes and Pathology

As described previously, it has been shown that emphysematous changes and basal fibrotic changes are a common finding in elderly patients, especially on CT. There are no normative values described in the literature, but age-related changes are usually described to be moderate. So, it is obvious that differentiation may be difficult to early changes in chronic obstructive lung disease or interstitial lung disease. 

For example, the finding of a moderate basal lung fibrosis may be due to age-related changes or findings of interstitial lung disease (usual interstitial pneumonia (UIP) or nonspecific interstitial pneumonia (NSIP)), which can be found along with autoimmune disease or idiopathic interstitial lung disease. A differentiation is important as the latter two need specific treatment in opposition to age-related changes. Therefore, close correlation between the morphological extent of the fibrotic changes, clinical history (i.e., known autoimmune disease), and observation of associated changes is crucial. Extensive changes as well as marked honeycombing or traction bronchiectasis are unlikely to be only age-related associated signs like ground glass opacities which have not been reported with age-related changes but may be due to congestive heart failure or infection. Correlation with preexisting imaging should be performed to assess disease progression.

The finding of senile emphysema is usually not accompanied by the clinical findings of chronic obstructive pulmonary disease like cough and sputum production. 

In some cases, differentiation may be not possible and follow-up imaging is needed. 

## 6. Pathologies with a Special Relevance in the Geriatric Population

### 6.1. Pneumonia

Pneumonia still is one of the leading causes of death from infection and is most commonly at the extreme of ages, that is, in the very young and in the elderly population [22]. In elderly patients, the immune system is often compromised. Beside an age-related decrease in immune activity, there are medications altering immune function. For example, long-term use of systemic corticosteroids in rheumatic disease significantly increases the risk of severe pneumonias with need for hospitalization [23]. Clinically, pneumonia can be divided into typical or atypical presentation, and in accordance to history in community acquired, nosocomial or infections in the immunocompromised. The most important imaging tool is conventional chest radiography. The role of radiography is to detect or rule out infiltrates, to show the extent of disease and possible complications, and to show response to treatment [24]. If pneumonia is suspected in an immunocompromised patient, a negative X-ray is not adequate to rule out infection and a CT should be advocated. Complications like empyema or abscesses are shown superiorly by CT [24]. There is a considerable overlap in the radiological morphology due to different pathogenic agents, so the morphological type of pneumonia is only a weak indicator of certain pathogens [25]. But in synopsis with clinical history and findings, chest X-ray will restrict the spectrum of possible pathogens and guide the calculated use of antibiotics. It has to be stressed that without clinical information, the differentiation between infectious infiltrates and other consolidating lung processes like cryptogenic organizing pneumonia is not possible [24]. If there are persistent infiltrations, bronchioalveolar carcinoma, now known as lepidic type of adenocarcinoma, should be included in the differential diagnosis. Clearance of pneumonic infiltration in the elderly takes usually more time. It has been shown that 15% of elderly patients still showed radiographic abnormalities beyond 3 months. Delayed clearance could be correlated to existing comorbidity [26]. We recommend a minimum interval of 3 months for the follow-up X-ray to to rule out preceeding malignant changes. Attention should be paid to the reactivation of tuberculosis. Many elderly patients are showing posttuberculotic changes on imaging. In reactivated pulmonary tuberculosis, patchy consolidations in the upper lobes or the superior segments of the lower lobes are the most common finding. Cavitations with a predilection in the upper lung zones can be found in up to 45% of patients [27]. 

### 6.2. Aspiration Pneumonia

**Figure 4:**
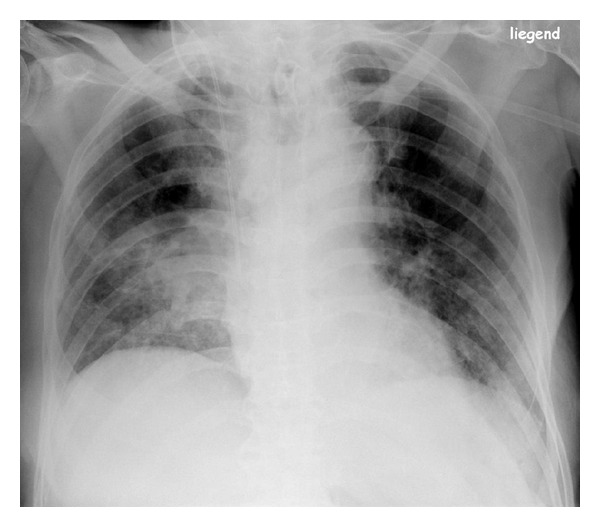
Consolidation in the right lower lobe due to aspiration in an 85-year-old patient (aspiration was confirmed using fluoroscopy).

Oropharyngeal contents or gastric acid which is misdirected to the lower airways can cause severe inflammation. In addition to the chemical pneumonitis pathogens from the oral flora, reaching the lower respiratory tract may cause difficult-to-treat bacterial pneumonia [28]. It has been shown that even healthy elderly people are swallowing more slowly than younger persons and the cough reflex is impaired. This may lead to pharyngeal colonisation with pathogenic bacteria [29, 30]. Aspiration of small amounts is common during sleep even in healthy young adults [31]. In conclusion to this, it seems that the amount of aspiration and/or colonization of the pharynx or gastric content by bacteria is important. It should be kept in mind that proton pump inhibitors and H_2_ antagonists cause an increase in gastric pH which supports bacterial colonisation of the stomach. In everyday practice, the major cause of dysphagia is stroke and Parkinson's syndrome [28]. In a study by Nakagawa et al. [32], in the followup after stroke, 24% of patients with dysphagia developed pneumonia within one year. In contrast, none of the stroke patients without dysphagia developed pneumonia. Radiologically, recurrently found infiltrates involving the right lower lobe or the upper lobes in elderly patients should raise the suspicion of aspiration pneumonia (Figure 4).

**Figure 5:**
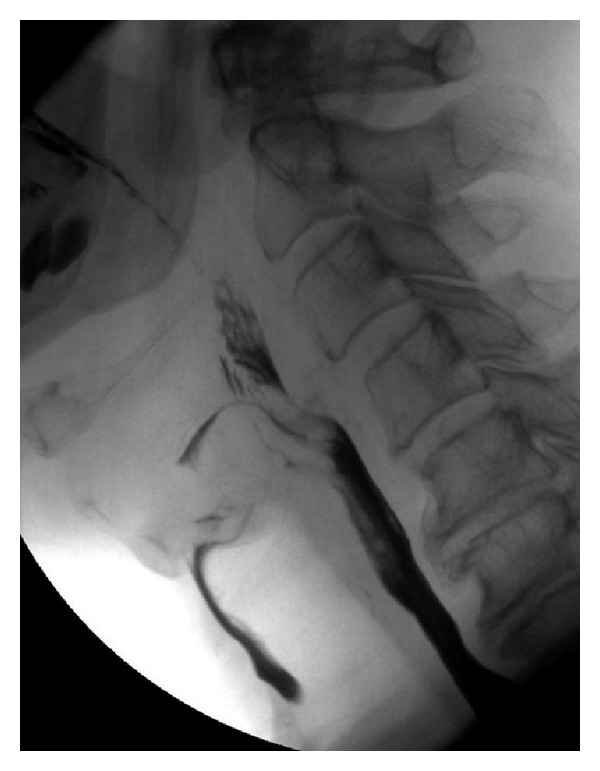
Spot view from a fluoroscopic examination in a 76-year-old patient after stroke showing aspiration.

 If aspiration is suspected, we perform a fluoroscopic swallowing study with the use of barium in different formed boluses (thick liquids, semithick liquids, semisolid food, or solid food). This may guide dietary modifications, which are the most common management approach [28]. A commonly found phenomenon in elderly patients is laryngeal penetration, which means that small amounts of contrast media are entering the larynx. Only if the barium passes, the glottis aspiration can be diagnosed (Figure 5). 

Another problem to be kept in mind is reflux in patients with percutaneous gastroenterostomy feeding tubes. In these patients, reflux may ultimately lead to aspiration. The diagnostic approach of choice is also fluoroscopy. The change to a jejunal position of the tip of the feeding tube can solve this problem, but it has to be kept in mind that jejunal tubes are easily congested making the clinical handling of these patients problematic. At last, the side effects of medications should be remembered. Neuroleptics may cause dyskinesia of the muscles needed for swallowing with consecutive aspiration. In one study, the use of neuroleptic medication was associated with a higher risk of aspiration pneumonia [33].

### 6.3. Lung Changes with Congestive Heart Failure

**Figure 6:**
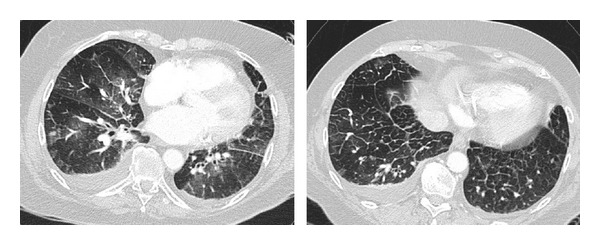
Computed tomography in 77-year-old patients showing signs of congestive heart failure with ground glass opacities, smooth thickening of interlobular septae, and bilateral effusions.

Cardiac disease, especially left heart failure, is a major differential diagnosis for dyspnea in the elderly. With pulmonary venous, hypertension hydrostatic edema of the lungs occurs with a well-known appearance on conventional chest X-ray: cranialization of the pulmonary blood flow, increased vascular and interstitial markings with Kerley B lines, peribronchial cuffing, heart enlargement, and pleural effusions. With progression, alveolar edema occurs which in most cases can be differentiated from edema of noncardiogenic causes, like renal edema or capillary permeability edema (e.g., ARDS) [34]. From our experience, the diagnosis of early stages of congestive heart failure may be complicated by concomitant fibrotic changes. Correlation with already existing images or serial imaging will help to solve this problem. In patients with emphysema due to chronic obstructive pulmonary disease (COPD) the distribution relies on the remaining intact parenchyma, so atypical findings are common. Pulmonary edema in the right upper lobe can occur in patients with severe mitral regurgitation. Pulmonary venous hypertension has typical features on CT imaging (enlargement of the upper lobe pulmonary vessels, thickening of the bronchial walls, diffuse smooth thickening of the interlobular septae, and ground glass opacities accompanied by effusions and heart enlargement, Figure 6) [35]. 

In particular, in elderly patients with dyspnea undergoing CTPA for pulmonary embolism, we often find signs of congestive heart failure

### 6.4. The Problem of Overlapping Pathology in COPD/Congestive Heart Failure

Beside the difficulty in discriminating age-related changes from pathology, there is the problem of clinical overlapping pathology with multimorbidity. With respect to imaging of the lung, the distinction between heart failure and COPD is a major concern. Up to 50% of patients with heart failure have concomitant COPD, and in most studies the prevalence was around 20%. On the other hand, about 20% of patients with COPD also have signs of left heart failure [36]. Heart failure mimics any clinical sign of COPD and vice versa, like cough, breathlessness, and exercise fatigue. Lung function tests may be misleading, and there is no established laboratory marker for differentiation between these two diseases. Therefore, in the case of acute dyspnea, it is a clinical routine to order a chest X-ray for further differential diagnosis. The major problem is that signs of heart failure may be atypical and asymmetric according to the areas with preserved normal pulmonary structure in patients with emphysematous lung changes in consequence to COPD. This may easily be confounded with peribronchial infiltrations, which are common during exacerbations of COPD. For a systematic differentiation, it is important to look for signs of COPD first. Theoretically, two extreme forms of COPD may be constructed: “pure” chronic bronchitis and “pure” emphysema. In clinical practice, as can be seen on CT, a mixture of these components is found in almost all patients with COPD. The pure forms are showing distinct changes in imaging. With “pure chronic bronchitis,” there is the finding of a “dirty chest” with increased interstitial lung markings and bronchial wall thickening. With further disease, progression signs of right heart enlargement and pulmonary arterial hypertension can be found. The value of chest radiography in chronic bronchitis has undergone considerable debate, but most authors state that the findings of a “dirty chest” are insensitive and have the problem of low reproducibility and interobserver variability [37]. The imaging of emphysema with chest radiography has undergone less debate, because the signs of hyperinflation of the lungs are obvious and objective measurements can be made [37]. The lateral view is of special importance, and it shows widening of the retrosternal space (>2.5 cm) and the flattening of the diaphragm (the angle between the chest wall and diaphragm is becoming larger than 90 degrees). If hyperinflation is found, atypical forms of pulmonary edema should be expected and kept in mind. Patients with initial emphysematous changes or senile emphysematous changes are normally not showing signs of significant hyperinflation on conventional X-ray. Our observations suggest that the destruction of normal pulmonary vascularity in these early stages is not marked enough to show noticeable asymmetric edematous changes with congestive heart failure. Because of the flattened costophrenic angles and scarring changes, ultrasound is often needed to make the diagnosis of small pleural effusion in patients with emphysema.

### 6.5. Pulmonary Drug Toxicity

**Figure 7:**
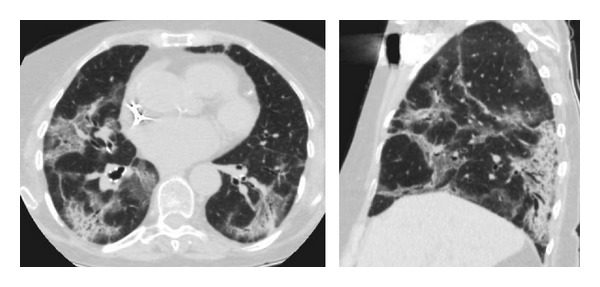
Drug induces lung changes with the use of amiodarone in an 81-year-old patient. Computed tomography shows the pattern of cryptogenic organizing pneumonia.

Pulmonary drug toxicity has recently received increased attention. Once believed to occur only with a few drugs, the list of causative agents is steadily growing. In a 2001 review, already about 150 causative drugs were mentioned and even more can be found in an internet database (PneumoTox) [38, 39]. The incidence is unclear, because systematic studies are lacking [40]. Age is not a risk factor per se, but as an effect of their multimorbidity, elderly people often take a variety of drugs. So, they are exposed to a wider range of possible causative agents and drug interactions (e.g., degradation via similar enzymatic mechanisms) which may cause an enhanced pulmonary toxicity [40]. If pulmonary drug toxicity is suggested or is a potential differential diagnosis, imaging with high resolution chest CT should be performed because of its superior sensitivity over plain radiography [41]. On imaging, common forms of toxic changes are fibrosing alveolitis (with a pattern often resembling findings in nonspecific interstitial pneumonia), predominantly subpleural consolidations (resembling cryptogenic organizing pneumonia or eosinophilic pneumonia), and in the more acute setting hypersensitivity reactions with imaging findings ranging from ground glass opacities and alveolar consolidations to severe diffuse alveolar damage indistinguishable from ARDS [38, 40] (Figure 7). 

Different clusters of drugs according to the radiological presentation have been proposed [39], but in general, the most important point is to consider the possibility of drug-induced lung disease in the elderly. 

### 6.6. Incidental Pulmonary Embolism

With the evolution of multislice CT, incidental PE has been shown to be an incidental finding in up to 6% of inpatients undergoing imaging of the chest with CT [42]. Incidental PE is more common in patients with known malignancy. Interestingly, in a study by Ritchie et al. [42], an increased prevalence with age was found. It is known that elderly people have a higher incidence of thromboembolic disease (symptomatic as well as asymptomatic). This may be explained by an elevated incidence of risk factors such as malignancy or immobility. In most cases, these incidental PEs are found on the subsegmental level. The clinical significance of incidental PE is unclear. As reviewed by Desai, currently available data suggests that even without treatment, mortality is not elevated [43]. Some authors argue that the lung acts as a filter, and the clearance of small emboli is a physiological process [44].

### 6.7. The Problem of Pulmonary Nodules

With the development of thin-section helical CT, the detection of small nodules, especially when using maximum intensity projections, has become routine. On chest X-ray, pulmonary nodules could be found in about 0.2% of patients [45]. In contrast, with multislice CT especially in lung cancer screening studies, the majority of patients showed pulmonary nodules [45]. There is a wide differential diagnosis, and the vast majority (over 80%) are granulomas or intrapulmonary lymph nodes with another 10% being hamartomas [46]. Morphologically, only benign forms of calcifications are a clear sign of nonmalignant nodules, these include complete, central, or popcorn-like calcifications. As the chance of malignancy increases with size, this is the major criterium for the need of further assessment and is central part of current guidelines [47]. Recently, special attention has been paid to the subset of subsolid nodules, because of the close correlation to the spectrum of adenocarcinoma dedicated guidelines by the Fleischner Society have been proposed. If possible, the comparison with older X-ray images is recommended as a large portion of nodules can be detected retrospectively, and a constant size over 2 years indicates benignancy [46]. In daily practice, small solid nodules are found in the majority of elderly patients. In our department, the following strategy is used in these cases: first we are looking for morphological signs of benignancy: benignant forms of calcifications, the presence of fat, the configuration of typical intrapulmonary lymph nodes, and cluster-like appearance in bronchiolitis with the typical “tree-in-bud” pattern. If none of these previously mentioned applies to the nodules, we are using adjusted guidelines of the Fleischner Society; that is, prolonged follow-up intervals (minimum 6 months) are recommended in close correlation with the clinical state of the patient [47]. It is important to remember that even in patients with known malignancy only a small portion of nodules smaller than 10 mm are in fact metastasis [48]. Therefore, follow-up imaging is also the method of choice in oncologic elderly patients with small pulmonary nodules. 

### 6.8. Trauma

The increasing risk of falls with ageing is an everyday topic in geriatric medicine. Ojo et al. studied the type of injuries with falls in elderly people and found chest injuries in 6.9% of patients [49]. In this group, the vast majority suffered from rib fractures (86%). The primary imaging test in suspected rib fracture is radiography, but even with dedicated oblique views, it has been reported that up to 50% of fractures are missed. In our department, with minor blunt trauma, we are performing a single oblique view of the affected side of the chest together with a standard radiography of the lung (single view, p.a.) to search for complications of the trauma (effusion, lung contusion, and pneumothorax) [50]. If there are uncertainties or there is major trauma, CT is the imaging of choice. Ultrasound has shown a high sensitivity for diagnosing rib fractures, but its use is time consuming and operator dependent. It may be reserved for selected cases, for example, further workup of suspected rib fracture in minor chest trauma despite negative radiographs [50]. 

## 7. Teaching Points/Conclusion

- The basic examination of the lung is chest radiography. If further workup is needed, chest CT should be performed. To minimize motion artifacts due to breathing, a caudocranial scan direction is recommended. If there are still motion artifacts hindering interpretation add some classical HR-CT scans.
- Common age-related changes include basal fibrotic changes, senile emphysema, and progressive calcification of the airways and rib cage.
- In particular, age-related fibrotic changes may be difficult to differentiate from early fibrotic changes with UIP/NSIP. Extensive changes as well as marked honeycombing, traction bronchiectasis, and ground glass opacities are unlikely in “pure” age-related changes.
- Resolution of pneumonic infiltrations is slower in the elderly, therefore recommend follow-up imaging after 3 months.
- If aspiration is suspected, fluoroscopic examinations may establish diagnosis.
- Congestive heart failure may show an atypical or asymmetric pattern in patients with preexisting lung disease which always includes heart failure in the differential diagnosis of dyspnea.
- Think of pulmonary drug toxicity.
- Follow-up imaging is usually the appropriate management strategy with pulmonary nodules.
- If in doubt, look out for existing radiographs for comparison.

## References

[1] United Nations Commission on Population and Development. 42nd Session: programme implementation and future work of the secretariat in the field of demographic trends.

[2] Statistisches Bundesamt (2011). *Diagnosedaten der Patienten und Patientinnen in Krankenhäusern*.

[3] Morley JE, Perry HM, Miller DK (2002). Something about frailty. *Journals of Gerontology. Series A*.

[4] Torres SL, Dutton AG, Linn-Watson TA (2010). *Patient Care in Imaging Technology*.

[5] ACR Appropiateness criteria http://www.acr.org/Quality-Safety/Appropriateness-Criteria/.

[6] Toprak O, Cirit M (2006). Risk factors for contrast-induced nephropathy. *Kidney and Blood Pressure Research*.

[7] Cigarroa RG, Lange RA, Williams RH, Hillis LD (1989). Dosing of contrast material to prevent contrast nephropathy in patients with renal disease. *American Journal of Medicine*.

[8] Engelkemier DR, Tadros A, Karimi A (2012). Lower iodine load in routine contrast-enhanced CT: an alternative imaging strategy. *Journal of Computer Assisted Tomography*.

[9] Gossner J (2012). Feasibility of computed tomography pulmoary angiography with low flow rates. *Journal of Clinical Imaging Science*.

[10] Szucs-Farkas Z, Schibler F, Cullmann J (2011). Diagnostic accuracy of pulmonary CT angiography at low tube voltage: intraindividual comparison of a normal-dose protocol at 120 kVp and a low-dose protocol at 80 kVp using reduced amount of contrast medium in a simulation study. *American Journal of Roentgenology*.

[11] Sartori S, Tombesi P (2010). Emerging roles for transthoracic ultrasonography in pleuropulmonary pathology. *World Journal of Radiology*.

[12] Freeman LM, Stein EG, Sprayregen S, Chamarthy M, Haramati LB (2008). The current and continuing important role of ventilation-perfusion scintigraphy in evaluating patients with suspected pulmonary embolism. *Seminars in Nuclear Medicine*.

[13] Wielpütz M, Kauczor HU (2012). MRI of the lung: state of the art. *Diagnostic and Interventional Radiology*.

[14] Mueller PS, Hook CC, Fleming KC (2004). Ethical issues in geriatrics: a guide for clinicians. *Mayo Clinic Proceedings*.

[15] Verbeken EK, Cauberghs M, Mertens I, Clement J, Lauweryns JM, van de Woestijne KP (1992). The senile lung; Comparison with normal and emphysematous lungs. 1. Structural aspects. *Chest*.

[16] Sharma G, Goodwin J (2006). Effect of aging on respiratory system physiology and immunology. *Clinical Interventions in Aging*.

[17] Heinrich A (1941). *Alternsvorgänge im Röntgenbild*.

[18] Copley SJ, Wells AU, Hawtin KE (2009). Lung morphology in the elderly: comparative CT study of subjects over 75 years old versus those under 55 years old. *Radiology*.

[19] Lee KW, Chung SY, Yang I, Lee Y, Ko EY, Park MJ (2000). Correlation of aging and smoking with air trapping at thin-section CT of the lung in asymptomatic subjects. *Radiology*.

[20] Hochhegger B, Pontes de Mereiles G, Irion K (2012). The chest and ageing: radiological findings. *Jornal Brasileiro de Pneumologia*.

[21] Caskey CI, Zerhouni EA, Fishman EK, Rahmouni AD (1989). Aging of the diaphragm: a CT study. *Radiology*.

[22] Reynolds JH, McDonald G, Alton H, Gordon SB (2010). Pneumonia in the immunocompetent patient. *British Journal of Radiology*.

[23] Bernatsky S, Hudson M, Suissa S (2007). Anti-rheumatic drug use and risk of serious infections in rheumatoid arthritis. *Rheumatology*.

[24] Franquet T (2001). Imaging of pneumonia: trends and algorithms. *European Respiratory Journal*.

[25] Tarver RD, Teague SD, Heitkamp DE, Conces DJ (2005). Radiology of community-acquired pneumonia. *Radiologic Clinics of North America*.

[26] El Solh AA, Aquilina AT, Gunen H, Ramadan F (2004). Radiographic resolution of community-acquired bacterial pneumonia in the elderly. *Journal of the American Geriatrics Society*.

[27] Jeong YJ, Lee KS (2008). Pulmonary tuberculosis: up-to-date imaging and management. *American Journal of Roentgenology*.

[28] Marik PE, Kaplan D (2003). Aspiration pneumonia and dysphagia in the elderly. *Chest*.

[29] Nagatake T (2003). Aspiration and aspiration pneumonia. *The Japan Medical Association Journal*.

[30] Robbins J, Hamilton JW, Lof GL, Kempster GB (1992). Oropharyngeal swallowing in normal adults of different ages. *Gastroenterology*.

[31] Gleeson K, Eggli DF, Maxwell SL (1997). Quantitative aspiration during sleep in normal subjects. *Chest*.

[32] Nakagawa T, Sekizawa K, Nakajoh K, Tanji H, Arai H, Sasaki H (2000). Silent cerebral infarction: a potential risk for pneumonia in the elderly. *Journal of Internal Medicine*.

[33] Wada H, Nakajoh K, Satoh-Nakagawa T (2001). Risk factors of aspiration pneumonia in Alzheimer’s disease patients. *Gerontology*.

[34] Milne ENC, Pistolesi M, Miniati M, Giuntini C (1985). The radiologic distinction of cardiogenic and noncardiogenic edema. *American Journal of Roentgenology*.

[35] Storto ML, Kee ST, Golden JA, Webb WR (1995). Hydrostatic pulmonary edema: high-resolution CT findings. *American Journal of Roentgenology*.

[36] Hawkins NM, Petrie MC, Jhund PS, Chalmers GW, Dunn FG, McMurray JJV (2009). Heart failure and chronic obstructive pulmonary disease: diagnostic pitfalls and epidemiology. *European Journal of Heart Failure*.

[37] Müller NL, Coxson H (2002). Chronic obstructive pulmonary disease • 4: imaging the lungs in patients with chronic obstructive pulmonary disease. *Thorax*.

[38] Özkan M, Dweik RA, Ahmad M (2001). Drug- induced lung disease. *Cleveland Clinic Journal of Medicine*.

[39] http://www.pneumotox.com/.

[40] Camus P, Foucher P, Bonniaud P, Ask K (2001). Drug-induced infiltrative lung disease. *European Respiratory Journal*.

[41] Ellis JE, Cleverly JR, Müller NL (2000). Drug- induced lung disease: high resolution CT findings. *American Journal of Roentgenology*.

[42] Ritchie G, McGurk S, McCreath C, Graham C, Murchison JT (2007). Prospective evaluation of unsuspected pulmonary embolism on contrast enhanced multidetector CT (MDCT) scanning. *Thorax*.

[43] Desai SR (2007). Unsuspected pulmonary embolism on CT scanning: yet another headache for clinicians?. *Thorax*.

[44] Gurney JW (1993). No fooling around: direct visualization of pulmonary embolism. *Radiology*.

[45] Holin SM, Dwork RE, Glaser S, Rikli AE, Stocklen JB (1959). Solitary pulmonary nodules found in a community-wide chest roentgenographic survey; a five-year follow-up study. *American Review of Tuberculosis*.

[46] Beigelman-Aubry C, Hill C, Grenier PA (2007). Management of an incidentally found pulmonary nodule. *European Radiology*.

[47] MacMahon H, Austin JHM, Gamsu G (2005). Guidelines for management of small pulmonary nodules detected on CT scans: a statement from the Fleischner Society. *Radiology*.

[48] Hanamiya M, Aoki T, Yamashita Y, Kawanami S, Korogi Y (2012). Frequency and significance of pulmonary nodules on thin-section CT in patients with extrapulmonary malignant neoplasms. *European Journal of Radiology*.

[49] Ojo P, O’Connor J, Kim D, Ciardiello K, Bonadies J (2009). Patterns of injury in geriatric falls. *Connecticut Medicine*.

[50] Bhavnagri SJ, Mohammed T-LH (2009). When and how to image a suspected broken rib. *Cleveland Clinic Journal of Medicine*.

